# Genome-wide association study (GWAS) reveals the genetic architecture of four husk traits in maize

**DOI:** 10.1186/s12864-016-3229-6

**Published:** 2016-11-21

**Authors:** Zhenhai Cui, Jinhong Luo, Chuangye Qi, Yanye Ruan, Jing Li, Ao Zhang, Xiaohong Yang, Yan He

**Affiliations:** 1 National Maize Improvement Center of China, Beijing Key Laboratory of Crop Genetic Improvement, China Agricultural University, Beijing, 100094 China; 2 College of Biological Science and Technology, Shenyang Agricultural University, Shenyang, 110866 China; 3 College of Agronomy, Shenyang Agricultural University, Shenyang, 110866 China

**Keywords:** Maize (Zea mays), Husk, Genetic architecture, GWAS, SNPs

## Abstract

**Background:**

Maize (Zea mays) husk referring to the leafy outer enclosing the ear, plays an important role in grain production by directly contributing photosynthate and protecting ear from pathogen infection. Although the physiological functions related to husk have been extensively studied, little is known about its morphological variation and genetic basis in natural population.

**Results:**

Here we utilized a maize association panel including 508 inbred lines with tropical, subtropical and temperate backgrounds to decipher the genetic architecture attributed to four husk traits, i.e. number of layers, length, width and thickness. Evaluating the phenotypic diversity at two different environments showed that four traits exhibit broadly natural variations and moderate levels of heritability with 0.64, 0.74, 0.49 and 0.75 for number, length, width and thickness, respectively. Diversity analysis indicated that different traits have dissimilar responses to subpopulation effects. A series of significantly positive or negative correlations between husk phenotypes and other agronomic traits were identified, indicating that husk growth is coordinated with other developmental processes. Combining husk traits with about half of a million of single nucleotide polymorphisms (SNPs) via genome-wide association study revealed a total of 9 variants significantly associated with traits at *P* < 1.04 × 10^-5^, which are implicated in multiple functional categories, such as cellular trafficking, transcriptional regulation and metabolism.

**Conclusions:**

These results provide instrumental information for understanding the genetic basis of husk development, and further studies on identified candidate genes facilitate to illuminate molecular pathways regulating maize husk growth.

**Electronic supplementary material:**

The online version of this article (doi:10.1186/s12864-016-3229-6) contains supplementary material, which is available to authorized users.

## Background

In the past decades, the husk surrounding the ear of maize has gained extensive attentions due to its special characters, such as operating a partial C_3_ photosynthetic pathway in contrast to stem leaves [1], the proper tightness and coverage protecting the ear infected from diseases [2, 3], and directly or indirectly providing plentiful sources of anthocyanin and fibre for nutritional or industrial production [4, 5]. The husk leaf area is positively correlated with the amount of cell-wall components such as the hemicellulose and cellulose fractions [6]. The husk area of flint corn genotypes expands fast with greater capability to synthesize xylose and arabinose, leading to the synthesis of hemicellulose [7]. Several husk traits including the husk thickness [8], the husk layer number [9], the husk tightness [10], the husk moisture [11] and the whole growing period of husk were reported to be intimately associated with harvest grain moisture [12]. Despite significant achievements in physiological research, we still lack the fundamental knowledge about the genetic basis underlying husk development.

Plant organ growth is generally resulted from the combined activities of two cellular processes, cell division and cell expansion [13]. The systematic studies have demonstrated major cellular pathways integrated to regulate each process of organ growth [14]. In a specific manner or dependent on their crosstalk, plant hormones such as indole-3-acetic acid (IAA), cytokinins (CKs), Gibberellins (GAs), brassinosteroids (BRs), abscisic acid (ABA) and strigolactones (SLs) have long been recognized as endogenous regulators of plant development [15]. In addition, the spatial distribution of reactive oxygen species (ROS) has been shown to define different organ growing zone and regulate meristem size [16]. Moreover, diverse metabolic pathways like sugars (e.g., sucrose and hexoses) and minerals (e.g., nitrates and phosphates) are shown to be essential for organ growth [17, 18]. Furthermore, the plant secretory pathway has roles in regulating cell growth mainly through transporting and depositing cell wall-synthesizing enzymes and polysaccharides [19]. In maize, the mechanisms controlling the growth of numerous organs, particularly for leaf and ear, have been well interpreted, while we know nearly nothing about how husk morphogenesis is regulated at the molecular level [20, 21].

The mutant screening has proven as an efficient approach to identify ‘master regulator’ genes required for specific stage of plant development in maize [22]. However, the weakness of this approach is loss-of-function mutations normally resulted in extreme mutant phenotype [14]. Therefore, the relevance of these identified genes in determining natural diversity of organ morphology in natural populations has not been easy to decipher [21]. Based on linkage analysis, mapping quantitative trait loci (QTL) is a powerful mean to identify novel genes and allelic variants that determine phenotypic variability between parents, especially for quantitative traits [23, 24]. Nevertheless, the majority of QTL analyses have been limited to a small number of genotypes, which harbor only a small portion of the natural variation [25, 26]. Genome-wide association study (GWAS), which is based on genetic linkage disequilibrium (LD) in a panel including a large number of genotypes representing broadly natural variations, has been used as an alternative approach for exploring the molecular basis and identifying SNPs of complex quantitative traits [27–30]. In maize, GWAS has been successfully utilized to identify numerous candidate loci/genes controlling a serious of morphological or metabolic traits, such as drought tolerance [31], starch content [32], stalk cell wall components [33], plant height [34], herbivore-induced volatiles [35], male inflorescence size [36], shoot apical meristem size [37], etc.

In this work, we used the GWAS approach employing 543,641 SNPs, with minor allele of frequency (MAF) ≥ 0.05 in a maize association population, to interpret the phenotypic diversity and genetic basis of four primary factors (number of layers, length, width and thickness) for husk morphogenesis and their relationships with other agronomic traits. A series of candidate genes that are associated with husk growth were identified, providing a useful resource for further functional studies to understand molecular pathways involved in husk growth and development.

## Results

### Husk diversity and heritability

The association population in this study consist of a global collection of 508 diverse maize inbred lines and have been successfully used to dissect the genetic basis of several complex traits, including beta-carotene [38], oil content [39], flowering time [40] and drought tolerance [41].

**Fig. 1:**
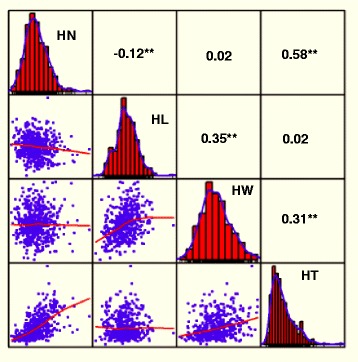
Frequency distributions and correlations of four husk traits. The plots on the diagonal line show the phenotypic distribution of each trait as indicated, the values above the diagonal line are Pearson-correlation coefficiencies between traits, and the plots below the diagonal line are the scatter plots of compared traits. **, *P* ≤ 0.01.HN, the number of husk layer; HL, the husk length; HW, the husk width; HT, the husk thickness

The field trial was conducted in two environments. All four husk traits followed a normal distribution with only husk thickness (HT) showing a slightly skew to the left (Fig. 1). Substantial variations among genotypes were observed for four husk traits (Table 1). Husk layer number (HN) was highly positive correlated with HT (*r* = 0.58, *P* ≤ 0.01) and negative correlated with husk length (HL, *r* = -0.12, *P* ≤ 0.01), husk width (HW) was highly positive correlated HL (r = 0.35, *P* ≤ 0.01) and HT (*r* = 0.31, *P* ≤ 0.01), while HN and HW, HL and HT were not correlated (Fig. 1). Significant variance components for genotype (G) and genotype × environment (G × E) interactions were observed for all four traits as shown in Table 1. However, G × E interactions represented relatively a small proportion of the total variance (Table 1). In addition, broad-sense heritability estimates were calculated, and the results showed moderate heritability for all three traits with HN(*h* ^*2*^ = 0.64), HL (*h* ^*2*^ = 0.74), HW (*h* ^*2*^ = 0.49) and HT (*h* ^*2*^ = 0.75), indicating that the main proportion of the phenotypic variations in husk phenotypes are derived from genetic factors, and suitable for further association mapping (Table 1).

**Table 1:** Phenotypic performance, variance component and broad-sense heritability of three husk traits

Trait^a^	Means ± SD (cm)	Range (cm)	Variance component^b,c^			*H* ^2d^
			G	E	GxE	
HN	9.67 ± 0.71	7.08–15.15	7.48**	9.34**	2.72**	0.64
HL	20.13 ± 1.19	13.71–30.80	27.01**	0.33	7.49**	0.74
HW	8.29 ± 0.96	5.52–11.50	8.16**	376.22**	4.15**	0.49
HT	2.12 ± 0.37	0.95–6.30	3.08**	133.72**	1.12**	0.75

^a^ *HN* husk number, *HL* husk length, *HW* husk width, *HT* husk thickness

^b^G and E indicate genotype and environment, respectively, and G × E indicate interaction of G and E

^c^*Significant at *P* ≤ 0.05; **Significant at *P* ≤ 0.01

^d^Family mean-based broad-sense heritability

**Fig. 2:**
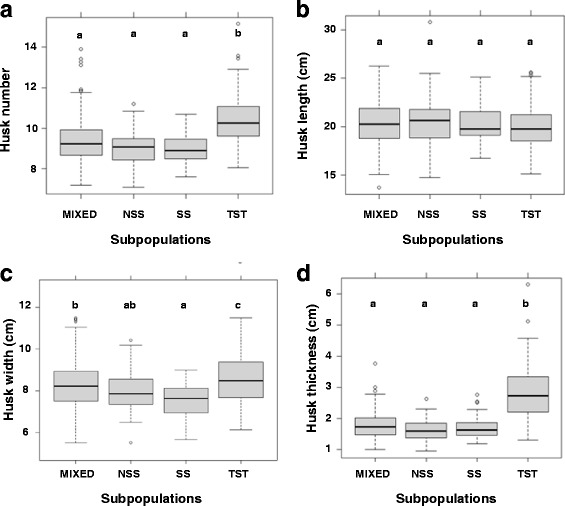
Boxplot of husk traits distribution in different subpopulations. Analysis of variance (ANOVA) was applied to examine the difference of traits among subpopulations. Different numbers indicate statically significant difference at *P* ≤ 0.05. No. of inbred lines included in each subpopulation are 215, 70, 27 and 196 for MIXED, NSS, SS and TST, respectively. **a** HN; **b** HL; **c** HW; **d** HT

The association panel used in this study can be divided into three subpopulations and one mixed group, which are termed by SS, NSS, TST and MIXED, respectively [42]. SS and NSS subpopulations are of temperate origin, and TST subpopulation is of tropical or subtropical origin while MIXED subpopulation encloses inbred lines which were not accurately assigned into the aforementioned three subpopulations based on the phylogenic analysis [42]. To investigate the effect of population structure on husk phenotypes, the phenotypic variations of husk traits were compared between different subpopulations. For HN, an increased mean in TST subpopulation compared to SS and NSS were observed, suggesting that maize inbred lines from tropical/subtropical origin tend to have more husk layers (Fig. 2a). For HL, no any significant difference was observed, indicating that population structure has no imposed effect on this trait (Fig. 2b). For HW, an increased mean and scale in TST subpopulation compared to SS and NSS were observed, suggesting that maize inbred lines from tropical/subtropical origin tend to have wider husk (Fig. 2c). The most significant variance were observed for HT, where the magnitude in TST subpopulation were remarkably larger than SS and NSS, indicating that maize inbred lines from tropical/subtropical origin are generally thicker (Fig. 2d). In summary, the husk traits show broad variations which are subject to genetic regulation and have dissimilar characters according to genetic backgrounds.

### Correlation analysis of maize husk phenotypes with other agronomic traits

The nature of husk as an integral part of maize ear prompts us to investigate how husk morphology is coordinated with other agronomic traits. To achieve this aim, the Pearson-correlations were calculated after comparing four husk traits with 17 agronomic traits which previously measured in the same association panel, including seven morphological traits, i.e. plant height (PH), ear height (EH), ear leaf width (ELW), ear leaf length (ELL), tassel maximum axis length (TMAL), tassel branch number (TBN), leaf number above ear (LNAE); seven yield traits, i.e. ear length (EL), ear diameter (ED), cob diameter (CD), kernel number per row (KNPR), cob grain weight (GW), cob weight (CW), kernel width (KW); three maturity traits, i.e. days to anthesis (DTA), days to silking (DTS) and days to heading (DTH) [43].

**Fig. 3:**
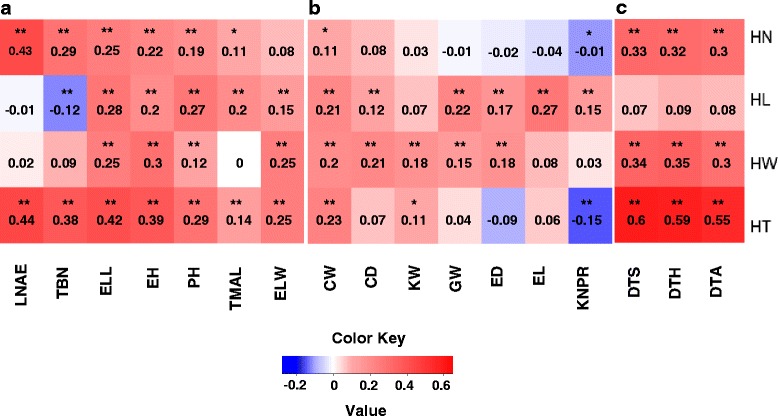
Correlation coefficients of husk phenotypes with other agronomic traits based on BLUP value. *Significant at *P* ≤ 0.05; **significant at *P* ≤ 0.01

The Best Linear Unbiased Prediction (BLUP) was calculated from the random effects of phenotypic data to represent unbiased mean estimates. All types of husk phenotype exhibited remarkably positive correlations with the other morphological traits. The exceptions are ELW for HN, LNAE for HL, LNAE, TBN and TMAL for HW (Fig. 3a). The most significant correlations were present between HN and LNAE, and between HT and LNAE (Fig. 3a). The only significantly negative correlation was observed between HL and TBN (Fig. 3a). Similarly, husk phenotypes showed intimate correlations with many aspects of yield trait. HN was positively correlated with CW and negatively correlated with KNPR (Fig. 3b). HL and HW are positively correlated with nearly all features of yield traits with the only exceptions, which were KW for HL as well as EL and KNPR for HW (Fig. 3b). For HT, it displayed positive correlations with CW and KW, and negative correlation with KNPR (Fig. 3b). Moreover, HN, HW and HT exhibited strongly positive correlations with all the parameters reflecting maize maturity (Fig. 3c).

### Genome-wide association analysis (GWAS)

**Fig. 4:**
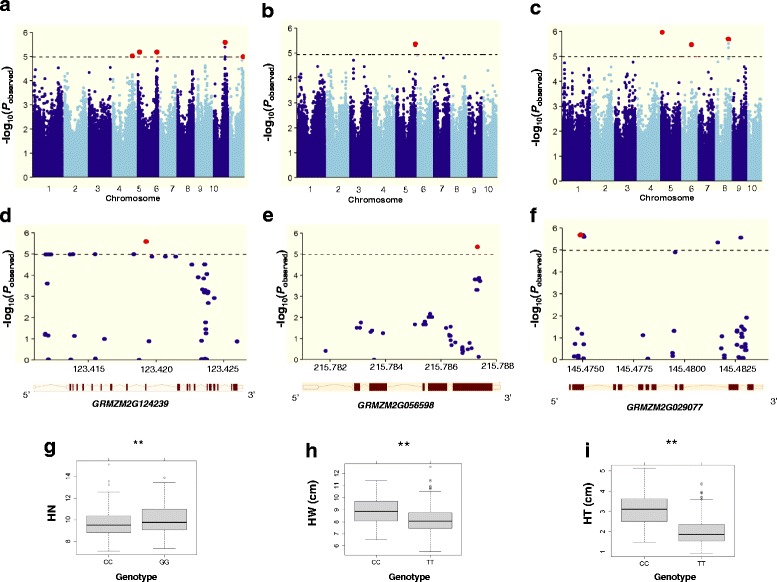
GWAS-derived Manhattan plots showing significant *P*-values associated with husk traits using MLM and association mapping results, genomic locations and allele effects of significant SNPs located around representative genes for husk traits. Each dot represents an SNP. The horizontal dashed blue line represents the Bonferroni-corrected significant threshold 1.04 × 10^-5^. Nine unique SNPs that met this level are enlarged and marked with red dots. **a** HN; **b** HW; **c** HT; **d**–**f** Regional plots showing association mapping results for SNPs located around *GRMZM2G124239* (**d**), *GRMZM2G029077* (**e**), *GRMZM2G056598* (**f**); **g**– **i** Allele effects of the most significant SNPs for husk traits. *Significant at *P* ≤ 0.05; **significant at *P* ≤ 0.01, **g** *GRMZM2G124239*, **h** *GRMZM2G029077*, **i** *GRMZM2G056598*

To minimize the effect of environmental variation, phenotypic BLUP values across two environments were used for association studies. GWAS was performed using a mixed linear model (MLM) and both kinship relationship (K matrix) and population structure (Q matrix) were taken into account to avoid spurious associations (See Methods) [44]. In total, we identified 5, 1 and 4 SNPs significantly associated with HN, HW and HT, respectively (Fig. 4; Table 2). The percentage of phenotypic variation explained by the identified SNPs (R^2^) for HN, HW and HT were 11.2, 4.9 and 21.4%, respectively (Table 2). In contrast, at this threshold, none of SNPs was detected to be significantly associated with HL. Moreover, GWAS was also performed using the general linear model (GLM), which identified 9, 25, 23 and 10 SNPs significantly associated with HN, HL, HW and HT, respectively (Additional file 1: Figure S1; Additional file 2: Table S1). Some SNPs were present in both methods, including 3 for HN and 3 for HT, respectively.

**Table 2:** SNPs, chromosomal position and candidate genes significantly associated with three husk traits identified by GWAS using MLM method

Trait	SNP	Chr	Position (bp)	Allele^a^	R^2^(%)^b^	MAF^c^	*P*-value	Gene	Gene interval (bp)	Annotation	Pathway^d^
HN	chr4.S_205554186	4	205554186	A/C	2.6	0.06	9.40E-06	GRMZM2G016447	205553899-205557585	Unknown	n.d
HN	SYN10582	5	17966191	G/A	0.2	0.19	6.43E-06	GRMZM2G097364	17965846-17977924	Unknown	n.d
HN	chr5.S_203403202	5	203403202	C/A	1.9	0.31	6.41E-06	GRMZM2G542272	203402576-203403244	Unknown	n.d
HN	chr9.S_123419252	9	123419252	C/G	4.1	0.18	2.57E-06	GRMZM2G124239	123411699-123426029	Vacuolar protein sorting-associated protein 52	Cellular trafficking
HN	chr10.S_143066093	10	143066093	C/G	3.7	0.08	1.00E-05	GRMZM2G396846	143066059-143068062	Global transcription factor group E7	Transcriptional regulation
Total^e^					11.2						
HW	chr5.S_215787314	5	215787314	T/C	4.9	0.18	4.51E-06	GRMZM2G056598	215781414-215787413	Chromatin remodeling 4	Transcriptional regulation
Total^e^					4.9						
HT	chr5.S_11925326	5	11925326	C/T	8.2	0.06	1.06E-06	GRMZM2G157166	11925082-11925880	Oxysterol binding protein-related protein 1A	Metabolism
HT	chr6.S_106444300	6	106444300	C/T	7.2	0.06	3.37E-06	GRMZM2G404081	106425279-106427173	GDSL/SGNH-like acyl-esterase family	Metabolism
HT	chr8.S_145474885	8	145474885	T/C	11.5	0.09	2.08E-06	GRMZM2G029077	145474389-145483146	Adaptin family	Cellular trafficking
Total^e^					21.4						

^a^Major/minor allele, underlined bases are the favorable alleles

^b^Percentage of phenotypic variation explained by the additive effect of the single significant SNP

^c^ Minor allele of frequency

^d^ *n.d* means not denoted

^e^ Total percentage of phenotypic variation explained by all significant SNPs

The allelic effects of haplotype coordinated with significantly differential SNPs on husk phenotypes were investigated and the enlarged red dots in Fig. 4d − f indicate the representative loci for each trait. For HN, the selected SNP (C/G, *p*-value = 2.57E-06, R^2^ = 4.1%) locates in the eighth intron of *GRMZM2G124239* (Fig. 4d). The average HN for allele with C was 9.62, significantly lesser than allele with G (10.0, *p* ≤ 0.01, Fig. 4g). For HW, the selected SNP (T/C, p-value = 4.51E-06, *R* ^2^ = 4.9%) locates in the fifth exon of *GRMZM2G056598* (Fig. 4e) and belongs to non-synonymous with the transition from leucine (CTC) to phenylalanine (TTC). The mean HW for allele with T was 8.14 cm, significantly narrower than allele with C (8.91 cm, *p* ≤ 0.01, Fig. 4h). For HT, the selected SNP (T/C, p-value = 2.08E-06, *R* ^2^ = 11.5%) locates in the second exon of *GRMZM2G029077* (Fig. 4f) and belongs to non-synonymous with the transition from alanine (GCA) to valine (GTA). The average HT for allele with C was 3.05 cm, significantly thicker than allele with T (2.04 cm, *p* ≤ 0.01, Fig. 4i).

### Genes co-localized with significant GWAS SNPs

All of 9 SNPs significantly associated with husk traits identified by MLM are located in genic regions (Table 2). The genes underlying these SNPs were grouped into several functional categories, exemplified by 2 genes involved in metabolic pathways, 1 genes in secretory transport, 2 gene in transcriptional regulation and 1 gene in cellular transport (Table 2). In addition, 61 out of 67 significant SNPs identified by GLM are located in genic regions (Additional file 2: Table S1). The functional categories of these genes were exemplified by 15 genes involved in metabolic pathways, 12 genes in transcriptional regulation, 9 gene in signal transduction, 6 gene in cellular transport, 5 genes in Intracellular trafficking and 2 gene in the reduction-oxidation reaction (Redox) (Additional file 2: Table S1).

**Fig. 5:**
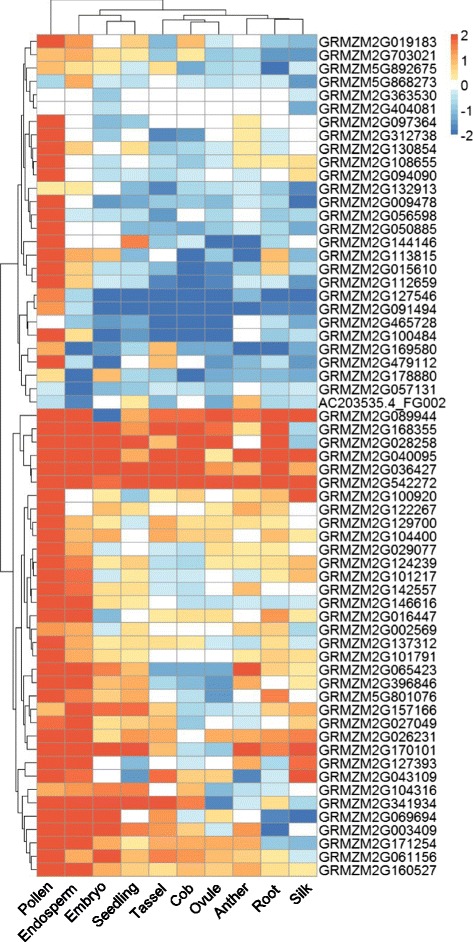
Heat-map of tissue-specific expression patterns of candidate genes identified by GWAS. The values used in the figure are the log_2_ transformed ratio of normalized PRKM count in husk relative to that in other tissues as shown at the bottom of each column. Columns and rows are ordered according to similarity (hierarchical cluster analysis at the top and left). The orange, white, and blue represent higher in husk than other tissues, close to other tissues, and lower than other tissues of a particular gene, respectively

To determine whether these genes denoted by significant SNPs were functional in the manner of tissue-specific expression, the *in silico* expression pattern was compiled using the published RNA-seq datasets from 11 different organs/tissues, including husk [45–51]. The dataset used in this analysis was listed in Additional file 2: Table S2. As shown in Fig. 5 and Additional file 2: Table S2, a set of candidate genes showed a tendency of higher expression in husk relative to other tissues. Subsequently, the quantitative real-time PCR (RT-qPCR) was conducted to validate the expression pattern of selective ten genes, including a total of nine genes present in MLM model and two genes in GLM model displaying relatively high expression in husk shown in Fig 5. It is noted that since *GRMZM2G404081* was not detectable by RT-qPCR, it is excluded from this analysis. As shown in Additional file 3: Figure S2, except of *GRMZM2G097364* and *GRMZM2G056598*, all the other eight genes exhibited more or less higher expression in husk relative to most of tissues tested. The highly expressions in husk further suggest the relevance of these candidate genes to act in husk morphogenesis.

## Discussion

Maize husk constitutes the leafy outer of ear and protects cob from dehydration and pathogen infection [2, 3, 10]. Therefore, the husk morphogenesis must be delicately coordinated with ear growth and development. In the past decades, due to the direct influence to grain production, the ear development in maize received extensive attentions [20, 52]. The genetic architecture of several ear-related traits has been illustrated in diverse maize populations [53–55], and several key genes involving in ear development have been characterized [20, 52]. In contrast, the genetic basis and the molecular pathways underlying husk morphology remain largely unknown. In this study, we interpret the natural variation and the associated genetic architecture of four primary husk traits in an association panel, and a set of putative candidate genes and pathways controlling husk development were revealed by performing GWAS analysis.

### Genetic basis of husk traits

All of four husk traits investigated in this study exhibited widely phenotypic variations with normal distribution. The genetic analysis shows that the heritability for HN, HL and HT and are higher than HW. The genetic contributions are significant, whereas the interaction of genetic and environmental effect was also significant for all traits.

The significant correlation is present between HL and HW, indicating that the growth and development of husk is coordinated in the aspect of length and width at a population level. The number of husk layer and the width of each layer constitute two basic factors which determine HT, and therefore, it is not surprising to observe that HT is positively correlated with HN and HW. In contrast, HL and HT are not correlated, indicating that the husk growth at the dimension of length has no effect on the thickness.

Maize is originated from tropical regions, and subsequently adapted into subtropical regions, and therefore, population structure may have imposed effects on maize morphology [56]. In this study, we observed that population structure has different effects on three husk traits. For HL, no significant difference was observed between subpopulations, indicating that the growth of husk in the dimension of length is parallel among each subpopulation. In contrast, the inbreds from tropical origin possess more layer of husk, as well as appear wider and thicker than those from subtropical origin, indicating that the shift of maize lines from tropical region to subtropical regions is accompanied with the characters of decreasing, narrowing and lessening husk growth.

### Coordination of husk morphology with other processes of plant development

As an integral part of ear, the growth and development of husk and ear would be intimately correlated. This is the case in our study. We observed a fairly close relationship of husk phenotypes with the corresponding features describing ear morphology. For instance, only HL, but not HN, HW or HT is highly correlated with EL. Meanwhile, HW is positively correlated with ED and CD, suggesting that the long or wide ears are endogenously enclosed by long or wide husk, respectively. In addition, all the husk traits show significantly positive correlations with CW, a parameter directly contributed by ear size, indicative of the fitness of husk establishment to the ears with large size. KNPR is positive correlated with HL, which is conceivable since the longer ears may create more space to allow additional kernels. Interestingly, however, KNPR showed negatively correlated HN and HT. This might be partly explained that HL and HN are negatively correlated, and the inbred lines with longer husk are companied with the reduction in the arithmetic formation of husk layer, consequently leading to the thinner HT.

As discussed above, the overall framework between husk and ear would be deliberately cooperated. Meanwhile, we found that the husk phenotypes also exhibited the substantially positively correlations with many aspects of maize morphological traits, indicating that the proper formation of husk morphology is intimately synchronized with the other aspects of plant growth and development. The most intriguing correlations were observed for HN, HW and HT with all the indexes reflecting maize maturity, indicating that the longer duration of growing period in maize is endowed with the features consisting of more layers of husk, as well as wider and thicker husk. Taken together, the correlation analyses between husk phenotypes with other agronomic traits reveal a number of both predictable and unpredictable associations, suggesting that husk morphology is coordinated with or affected by other processes of plant growth.

### Putative genes and pathways involved in husk morphogenesis

Plant organ formation consist of an initial phase of cell fate decision and cell division, leading to the construction of primary morphogenesis, then followed with a second phase of cell expansion and secondary morphogenesis [19]. A diverse range of genes are involved in the orchestration of organ formation by promoting or inhibiting component processes or pathways [14]. While the cellular pathway and its regulation attributing to ear development have been extensively studied in maize, entirely nothing is known about how husk morphogenesis is controlled at molecular levels [20, 21]. GWAS has been proved to be a powerful tool to rapidly discover prospective genes related to traits under investigation in crops [27–30]. In this study, we totally identify 9 genes significantly correlated with husk phenotype by MLM method and 61 genes by GLM method as well as. 6 genes are present in both methods. Functional annotation revealed that these candidate genes are mainly grouped into a few of functional groups, such as membrane vesicle trafficking, transcriptional regulation, redox and cellular transports, all of which have been reported to be critical for diverse processes of plant organ growth [57–60].

Intracellular trafficking of membrane-coated vesicles represents a fundamental process that regulates the flow of membrane materials among different endomembrane compartments inside and outside of the cell [61]. Key trafficking pathways consist of an inward flux of endocytic vesicles from the plasma membrane and an outward flux of exocytic vesicles to the plasma membrane [62, 63]. The major proteins underlying membrane trafficking include four phases of trafficking process, the components of which include vesicle coats, motors like myosin, adaptor complexes, SNARE proteins and Rab GTPases, and so on [64–67]. In Arabidopsis, several membrane trafficking-related genes have been shown to participate in diverse aspects of organ growth by affecting cytokinesis, a crucial procedure for successful cell division [68]. In this study, beside three genes encoding transmembrane protein, four genes directly involving in vesicle trafficking were identified, and fall into different phase of trafficking process. *GRMZM2G363530* encodes a subunit of coatomer, a protein complex required for Golgi non-clathrin-coated vesicles. *GRMZM2G703021* encodes a myosin protein, which helps to transport vesicles along a cytoskeletal track [69]. *GRMZM2G029077* encodes an adaptin protein, which mediate the formation of clathrin-coated vesicles. *GRMZM2G124239* encodes a homolog of the yeast *Vps52p/SAC2*, the homologs in yeast has been shown to function in a complex participated in retrograde trafficking of vesicles between the endosomal compartment and the trans-Golgi network [70]. In Arabidopsis, the disruption of *Vps52p/SAC2* led to the defect in pollen tube germination and growth [71]. With regard to the vital roles of intracellular trafficking in regulating plant organogenesis [72], the finding of these candidate genes implies that the intracellular trafficking pathway may have significant effects on the natural variations in husk traits.

Regulation of gene expression plays a central role in deciding the production of specific gene. Gene expression can be regulated at multiple levels, from chromatin organization, to DNA-RNA transcription initiation, to RNA processing, and to the post-translational modification of a protein [73–76]. We totally found 12 genes which are potentially acting in different levels of gene expression regulation, including a chromatin remodeling factor (*GRMZM2G056598*), a mediator for RNA polymerase II (*GRMZM2G160527*), 6 transcriptional regulators (*GRMZM2G104400*,*GRMZM2G396846,GRMZM2G057131*, *GRMZM2G130854*, *AC203535.4_FG002* and *GRMZM2G169580*), 4 RNA-binding proteins (*GRMZM2G061156*, *GRMZM2G104316*, *GRMZM2G142557* and *GRMZM2G122267*) and a RNA editing factor (*GRMZM2G071162*).

Metabolism is an indispensable part of a plant life cycle and contributes to a large part of plant phenotype performance [77, 78]. The disruptions of some certain metabolic pathways have profound influences on plant growth and development [79, 80]. Identical to other organs, husk growth must be a dynamic process that involves an interconnected series of metabolic pathways. Therefore, it is not surprising to identify a large number of candidate genes acting in diverse metabolic pathways associated with husk phenotypes. The representative pathways we identified include cytokinin (*GRMZM2G170101*), JA (*GRMZM2G040095*), sulfur (*GRMZM5G868273*), cellulose (*GRMZM2G178880* and *GRMZM2G002569*), flavonol (*GRMZM2G168355*), pectin (*GRMZM2G113815*), nucleotide (*GRMZM2G036427*) and glyoxylate (*GRMZM2G127546* and *GRMZM2G089944*). Cellulose synthase, encoded by a medium size of gene family in maize, have important roles in cell wall formation [81]. A previous study has shown that the mutation in one of the members, CslD, caused a substantial reduction in leaf width [82] Here, we identified two genes belonging to cellulose synthase and are significantly associated with HL and HW, respectively. Taken together, we speculate that the variation of these metabolic pathways in natural maize population may integrate together to control the diversity of husk morphology.

## Conclusions

The initial incentive of the present study is, until now, we lack any knowledge about genetic architecture and mechanism controlling natural variation in maize husk development. The results show that the husk morphological traits are moderately inheritable, showing a broad variation in a population containing 508 global diverse inbred lines genotyped by 543,641 polymorphic SNP markers. The GWAS demonstrated there are a number of genetic loci with small effect on regulating the natural variation in husk morphology, reflective of the complexity of husk development in maize. The candidate genes underlying these associated loci provide invaluable resource for the further study to functionally dissect the molecular network in regulating maize husk development, and the identification of SNP polymorphisms will be very useful for marker-assisted selection of husk traits in breeding program.

## Methods

### Association mapping panel and genotyping

The association panel consisted of 508 diverse lines, including 60 lines from the Germplasm Enhancement of Maize (GEM) project, 223 lines from the International Maize and Wheat Improvement Center (CIMMYT), and 225 lines from China. Most of the CIMMYT lines were of tropical or subtropical origin, whereas most lines from the USA and China were of temperate origin. To further examine the relatedness among 508 lines by *K* (the number of subpopulations based on the model), the maize panel was clustered into three clear subpopulations with 27 SS lines, 70 NSS lines and 196 TST lines; the remaining 215 lines were thus classified into a mixed subpopulation. Detailed information on 508 of these lines was described in two previous study [42, 83]. Less than ten lines didn’t germinate at each environment and were treated as missing data. All the lines were genotyped using the Illumina MaizeSNP50 BeadChip (Illumina) and 368 lines were genotyped by RNA-sequencing the developing kernels at 15 days after pollination [84]. The method of SNP projection and imputation were described in Yang et al. (2014) [43]. Totally about half of million SNPs were used in this study.

### Field experiments and phenotyping

All 508 lines of the association panel were planted at two different locations in China, which are Sanya, Hainan province in 2013 and Beijing in 2014. At each location, all the lines were planted in a single row plot with two replications using a complete randomized block design. At maturity, three husk traits were measured at the same time. Husk number was counted from the first layer of husk to the last. Husk length was measured at the 3^th^ layer of husk from top to bottom. Husk width was also determined by measuring the middle section of 3^th^ husk. Husk thickness was determined by measuring the total thickness by punching a disc from the interior to the exterior of husk layers. The method of how the phenotyping was conducted was diagramed in the supplemental Additional file 4: Figure S3. The data about 17 agronomic traits were collected by a previous study, including 7 morphological attributes (plant height, ear height, ear leaf width and length, tassel main axis length, tassel branch number, and leaf number above ear), 7 yield related traits (ear length and diameter, cob diameter, kernel number per row, 100-grain weight, cob weight, and kernel width), and 3 maturity traits (days to heading, anthesis, and silking) [43].

### Phenotype statistical analysis

ANOVA analysis of all husk traits in the association panel were performed by using the following mixed model: *y*_*ijk*_ = *μ* + *e*_*l*_ + *r*_*k(l)*_ + *f*_*i*_ + *(fe)*_*il*_ + *ε*_*lik*_, where *μ* is the grand mean of husk traits, *f*_*i*_ is the genetic effect of the *i*th line, *e*_*l*_ is environmental effect of the *l*th environment, *(fe)*_*il*_ is the interaction effect between genetic and environmental effects, *r*_*k(l)*_ is effect of replications within environments, and *ε*_*lik*_ is the residual error. The PROC MIXED procedure in SAS software (Release 9.1.3; SAS Institute, Cary, NC) was used to get the variance components of all husk traits. These variance components were used to calculate the broad-sense heritability as *h* ^*2*^ = *σ*_*g*_^*2*^/(*σ*_*g*_^*2*^ + *σ*_*ge*_^*2*^/*e + σ*_*ε*_^*2*^/r*e*) [85], where *σ*_*g*_^*2*^ is the genetic variance, *σ*_*ge*_^*2*^ is the interaction of genotype with environment, *σ*_*ε*_^*2*^ is the residual error, *e* and *r* represent the number of environments and replications in each environment.

### Genome-wide association mapping and phenotypic variance contribution of significant loci

The 543,641 SNPs (MAF ≥ 0.05) were selected for a GWAS by combining the data from two genotyping platforms (RNA-seq and SNP array) [25]. Association analysis for the 4 husk traits were conducted by the mixed linear model (MLM), taken both K and Q matrix into account to avoid spurious associations, presented in TASSEL V5.0 software package [86]. Considering of LD between SNPs in the genome-wide studies, the effective number of independent markers for the adjustment of multiple were used to obtain the *P* value thresholds [87]. The 95,742 markers in approximate linkage equilibrium with each other were found by PLINK [88] (the LD R^2^ threshold is 0.2), which was discussed and used by Mao et al. (2015) [89]. Then we used the uniform Bonferroni-corrected thresholds at α = 1 for MLM and α = 0.05 for general linear model (GLM) as the significance cutoffs as reported in the previous studies [39, 43, 89]. Therefore, the suggestive *P* value was computed by 1/n and 0.05/n (*n* = 95,742), and we obtained the *P* value were 1.04 × 10^-5^ for MLM and 5.2 × 10^-7^ for GLM as the final significance cutoff in the association analysis.

The contribution of SNPs to the phenotypic variance was estimated using anova() function in the R package. The R^2^ of each significant SNP after adjusting for the population structure effects, were calculated by the linear models: *Y = αX + βP + ε* (1) and *Y = βP + ε* (2).

For all SNPs of husk traits and estimate The total variance of all significant SNPs were calculated by the linear models: $\mathrm{Y}=\alpha {\displaystyle \sum_{i=1}^{\mathrm{m}} Xi}+\beta P+\varepsilon$ (3) and *Y = βP+ε* (2), where *Y* and *X* represent the phenotype and SNP genotype vectors, respectively; *P* is the matrix of three subpopulations (NSS, SS, TST); *α* is the SNP effect, *β* is the subpopulation effects, *ε* is the random effects.

### Annotation of candidate genes

The SNP with the most significance within the same LD block d (*r* ^2^ < 0.2) was selected to represent the locus. The physical locations of the SNPs were recorded according to the B73 RefGen_v2 (www.maizesequence.org). The corresponding genes were annotated by performing BLASTP search through NCBI website, and the candidate genes were assigned into different biological processes on the basis of the literatures describing the function of their homologs in other species or the knowledge in conserved domain database (CDD).

### Heat-map of candidate genes

Raw datasets of RNA-Seq from different maize tissues were downloaded from NCBI's Sequence Read Archive (SRA) database. The details about data sources were described in the Additional file 2: Table S2. FastQC (www.bioinformatics.babraham.ac.uk/projects/fastqc/) was initially run to examine the quality of RNA-Seq reads. Adapter and Low-quality reads were removed using fastx_clipper (parameters “-a GATCGGAAGAGCACACGTCTGAACTCCAGTCAC-l 30”) and fastq_quality_trimmer (parameters “-t 20 –l 30”) from the FASTX-Toolkit. RNA-Seq reads were aligned to the maize B73 reference genome (Zea_mays.AGPv3.28.dna.genome.fa) using the TopHat2 pipeline with the built-in Bowtie mapping program. The unique mapped reads were counted by htseq-count (HTSeq). To normalize the RNA-Seq data across the eleven samples from different maize tissues, we used the scaling normalization method provided in the edgeR package, based on a trimmed mean of M-values algorithm to compute the scaling factors according to the library size of each sample. After edgeR normalization, the RPKM values were averaged from replicates and used in the further analysis. The values used in the Fig. 5 are the log2 transformed ratio of normalized RPKM count in husk relative to other tissues. The values greater than +2 or less than -2 are adjusted to 2 or -2, respectively.

### RNA extraction and RT-PCR

RNA was extracted from different tissues using the RNeasy Plant Mini kit (Qiagen). cDNA was synthesized from 5 μg of total RNA using the ProtoScript® First Strand cDNA Synthesis Kit (New England Biolabs) following manufacturer’s instructions. RT-PCR reactions were performed as previously described [90] using primers listed in Additional file 2: Table S3. Transcript levels were estimated using the comparative CT method utilizing *UBQ1* as an internal control for data normalization. Data shown in Additional file 3: Figure S2 are averages of three independent experiments.

## Additional files

Additional file 1: Figure S1. GWAS-derived Manhattan plots showing significant P-values associated with husk traits using GLM. Each dot represents an SNP. The horizontal dashed blue line represents the Bonferroni-corrected significant threshold 5.2×10-7. (A) HN; (B) HL; (C) HW; (D) HT. (PPTX 163 kb)
Additional file 2: Table S1-S3. List of primers used in the study. (XLSX 44 kb)
Additional file 3: Figure S2. Relative expression pattern of 10 selective genes in husk versus other tissues verified using RT-qPCR . The expression in each tissue was first normalized using the UBQ1 (GRMZM2G409726). Y-axis: the relative expression of each gene in husk relative to other tissues as indicated (log2 scale). Data is shown as the mean ± SD of three independent experiments. (PPTX 71 kb)
Additional file 4: Figure S3. Diagram of phenotyping husk traits. (A) husk number; (B) husk length; (C) husk width; (D) husk thickness.. (PPTX 346 kb)

## Abbreviations

BLUP: Best Linear Unbiased prediction
CD: Cob diameter
CIMMYT: International Maize and Wheat Improvement Center
CW: Cob weight
DTA: Days to anthesis
DTH: Days to heading
DTS: Days to silking
E: Environment
ED: Ear diameter
EH: Ear height
EL: Ear length
ELL: Ear leaf length
ELW: Ear leaf width
G: Genotype
GEM: Germplasm Enhancement of Maize
GLM: General linear model
GO: Gene ontology
GW: Cob grain weight
GWAS: Genome-wide association study
HL: Husk length
HN: Husk number
HT: Husk thickness
HW: Husk width
K matrix: Kinship relationship
KNPR: Kernel number per row
KW: Kernel width
LD: Linkage disequilibrium
LNAE: Leaf number above ear
MLM: Mixed linear model
PH: Plant height
Q matrix: Population structure
QTL: Quantitative trait loci
R^2^: percentage of phenotypic variation explained by the identified SNPs
ROS: Reactive oxygen species
SNPs: Single nucleotide polymorphisms
SRA: Sequence read archive
TBN: Tassel branch number
TMAL: Tassel maximum axis length
