# Supplementary material for: Genome-wide association study (GWAS) reveals the genetic architecture of four husk traits in maize
Source: BMC Genomics. 2016 Nov 21;17:946. doi: 10.1186/s12864-016-3229-6 (PMC5117540; doi:10.1186/s12864-016-3229-6)
Supplement: Additional file 1: Figure S1. — GWAS-derived Manhattan plots showing significant P-values associated with husk traits using GLM. Each dot represents an SNP. The horizontal dashed blue line represents the Bonferroni-corrected significant threshold 5.2×10^-7^. (A) HN; (B) HL; (C) HW; (D) HT. (PPTX 163 kb) [file 12864_2016_3229_MOESM1_ESM.pptx]

## Slide 1
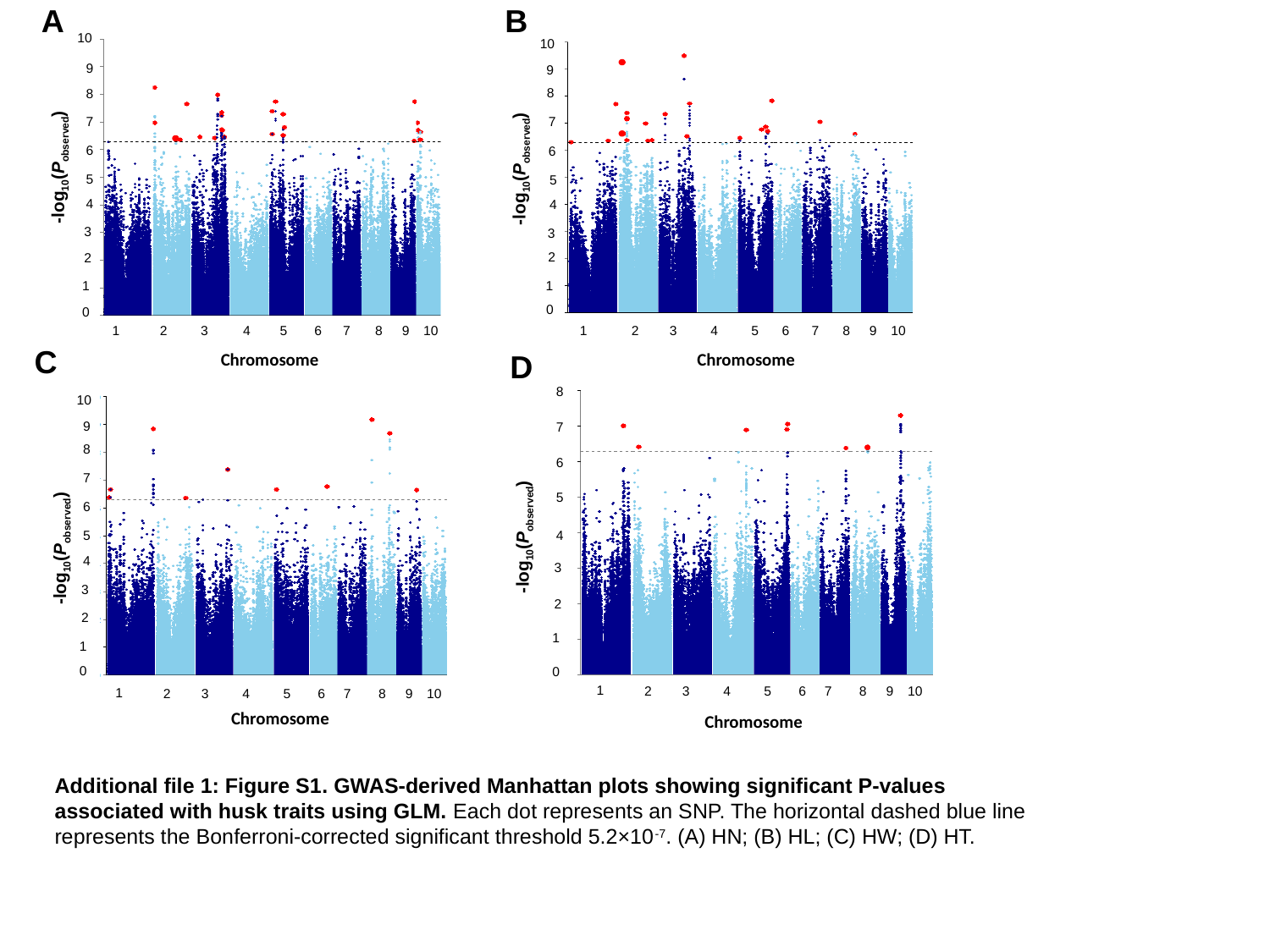

B
A
10
10
9
8
7
6
5
4
3
2
1
0
9
8
7
6
-log10(Pobserved)
-log10(Pobserved)
5
4
3
2
1
0
1
2
3
4
5
6
7
8
9
10
1
2
3
4
5
6
7
8
9
10
C
D
Chromosome
Chromosome
8
10
9
7
8
6
7
5
6
-log10(Pobserved)
4
5
-log10(Pobserved)
4
3
3
2
2
1
1
0
0
1
3
4
5
6
7
8
2
9
10
1
3
4
5
6
7
8
2
9
10
Chromosome
Chromosome
Additional file 1: Figure S1. GWAS-derived Manhattan plots showing significant P-values associated with husk traits using GLM. Each dot represents an SNP. The horizontal dashed blue line represents the Bonferroni-corrected significant threshold 5.2×10^-7^. (A) HN; (B) HL; (C) HW; (D) HT.
