# Supplementary material for: Genome-wide association study (GWAS) reveals the genetic architecture of four husk traits in maize
Source: BMC Genomics. 2016 Nov 21;17:946. doi: 10.1186/s12864-016-3229-6 (PMC5117540; doi:10.1186/s12864-016-3229-6)
Supplement: Additional file 3: Figure S2. — Relative expression pattern of 10 selective genes in husk versus other tissues verified using RT-qPCR . The expression in each tissue was first normalized using the UBQ1 (GRMZM2G409726). Y-axis: the relative expression of each gene in husk relative to other tissues as indicated (log2 scale). Data is shown as the mean ± SD of three independent experiments. (PPTX 71 kb) [file 12864_2016_3229_MOESM3_ESM.pptx]

## Slide 1
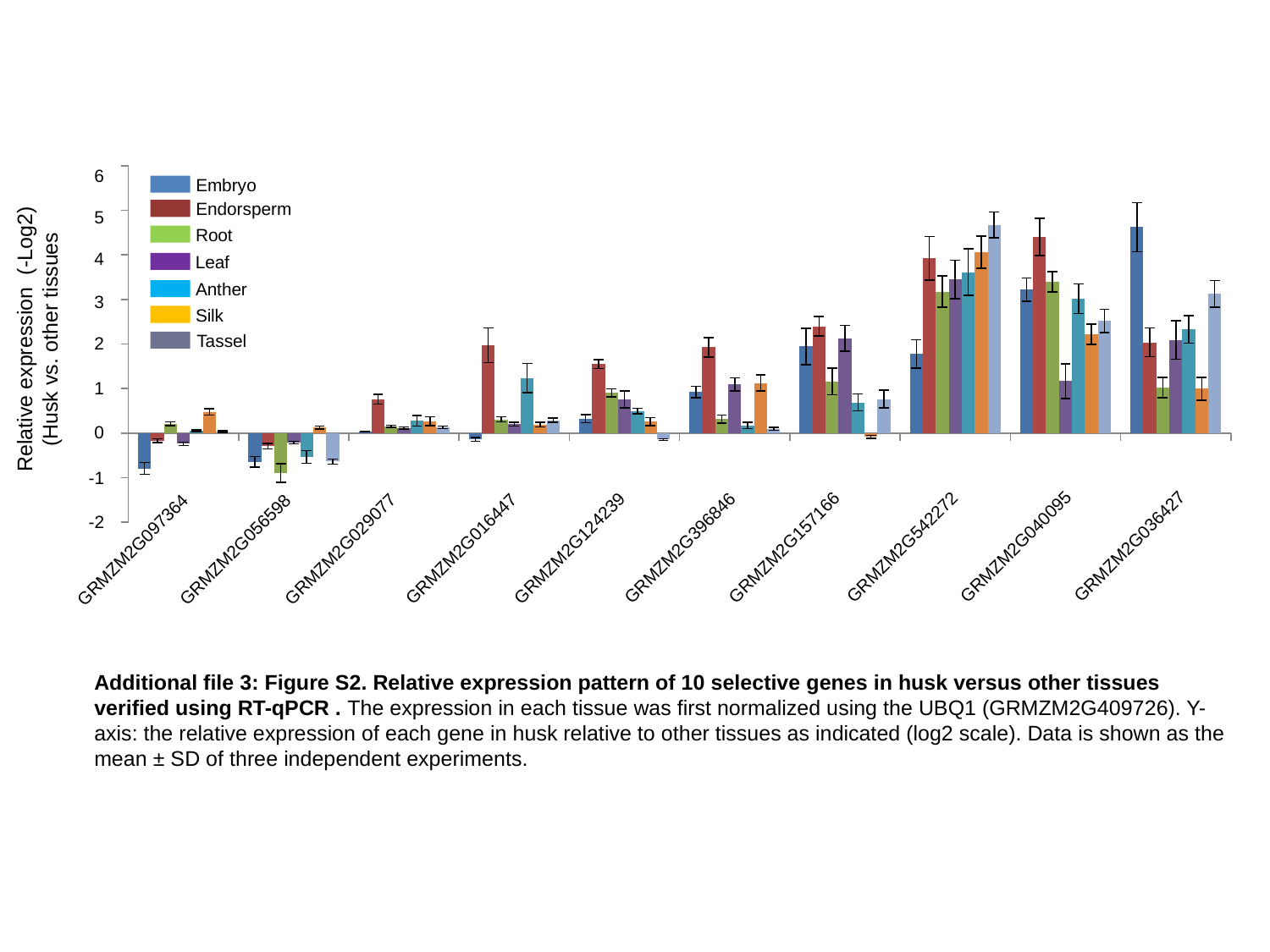

### Chart
| Category | | | | | | | |
|---|---|---|---|---|---|---|---|6
Embryo
Endorsperm
5
Root
4
Leaf
Anther
3
Silk
Relative expression (-Log2)
(Husk vs. other tissues
Tassel
2
1
0
-1
-2
GRMZM2G036427
GRMZM2G040095
GRMZM2G542272
GRMZM2G157166
GRMZM2G396846
GRMZM2G124239
GRMZM2G016447
GRMZM2G029077
GRMZM2G056598
GRMZM2G097364
Additional file 3: Figure S2. Relative expression pattern of 10 selective genes in husk versus other tissues verified using RT-qPCR . The expression in each tissue was first normalized using the UBQ1 (GRMZM2G409726). Y-axis: the relative expression of each gene in husk relative to other tissues as indicated (log2 scale). Data is shown as the mean ± SD of three independent experiments.
