# Supplementary material for: Genome-wide association study (GWAS) reveals the genetic architecture of four husk traits in maize
Source: BMC Genomics. 2016 Nov 21;17:946. doi: 10.1186/s12864-016-3229-6 (PMC5117540; doi:10.1186/s12864-016-3229-6)
Supplement: Additional file 4: Figure S3. — Diagram of phenotyping husk traits. (A) husk number; (B) husk length; (C) husk width; (D) husk thickness.. (PPTX 346 kb) [file 12864_2016_3229_MOESM4_ESM.pptx]

## Slide 1
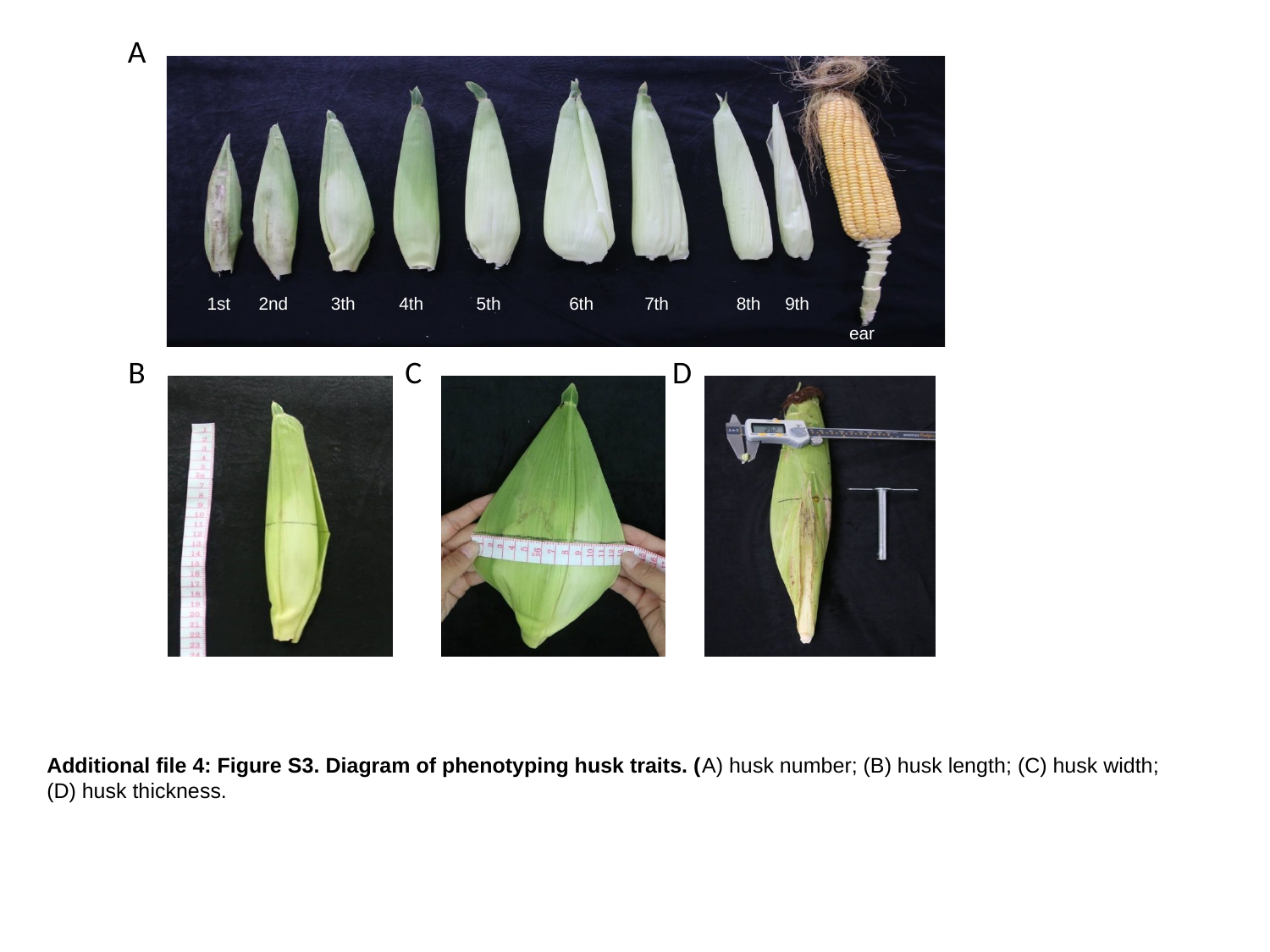

A
2nd
3th
4th
6th
7th
8th
9th
1st
5th
ear
B
C
D
Additional file 4: Figure S3. Diagram of phenotyping husk traits. (A) husk number; (B) husk length; (C) husk width; (D) husk thickness.
